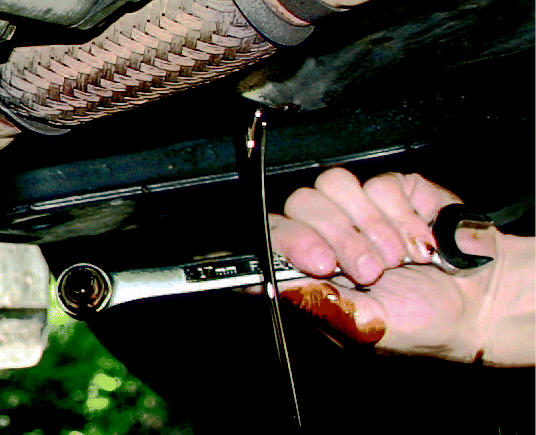# The Beat

**Published:** 2004-08

**Authors:** Erin E. Dooley

## Further Support for Sustainable Cities

In February 2004, representatives at the Asia and Pacific Leadership Forum adopted the Hong Kong Declaration on Sustainable Development for Cities. The document stems from Agenda 21, recently reaffirmed at the World Summit on Sustainable Development, and sets a goal of significantly improving the lives of at least 10% of the world’s estimated 1 billion slum dwellers. The declaration encourages cities to develop comprehensive strategies for not only economic development, but environmental protection as well, and notes the role that education and public health play in sustainable development. The declaration also notes the challenge that urban transportation poses to sustainable development, particularly in cities in the Asia/Pacific region.

## BBC ’Toons Tout Healthier Snacks

No longer will popular BBC cartoon characters like the Teletubbies and the Tweenies grace the labels of unhealthy snack foods. In April 2004, network officials announced the characters’ removal from labels of products with high sugar, salt, and fat contents, in response to growing concerns over children’s diets and obesity. The network will continue to license its characters for healthier foods including yogurt, pasta, and bread, as well as special-occasion treats like birthday cakes. The network is also planning to license the characters for a line of staple foods including fruit, vegetables, meat, milk, and dairy products. This new move follows a July 2003 decision by the network to end a sponsorship deal with McDonald’s using BBC characters.

## Fast Food Premieres

Director Morgan Spurlock has documented the impact the fast-food industry has on Americans’ waistlines in his film *Super Size Me*. With obesity affecting growing numbers of adults and children alike, Spurlock wanted to find out what was causing this epidemic. He interviewed people in 20 cities, from children eating at McDonald’s to the U.S. Surgeon General, and lived on nothing but fast food for an entire month while he made the movie, gaining 25 pounds and damaging his liver in the process. The website for the movie (**http://www.supersizeme.com/**) notes that each day 1 in 4 Americans visits a fast-food restaurant, and that most nutritionists recommend not eating fast food more than once a month.

## London Hits Volume Control

Noting that noise can affect a person’s speech, learning, and concentration, the mayor of London, England, has set forth a citywide plan for a quieter capital. The plan requires reductions by all sources of ambient noise, at all times of day—the three main priorities for the strategy are improving and maintaining road surfaces, securing a night-time aircraft ban over the city, and reducing noise through better planning and design of new housing. Ongoing incentives for alternative vehicles will also help decrease noise, as these vehicles are often quieter than their conventional counterparts. The London initiative comes ahead of a requirement that the entire country enact an ambient noise strategy by 2007.

## U.S. Signs Tobacco Treaty

In May 2004, the United States became the 108th country to sign the WHO’s international treaty on tobacco control, which outlines a plan of action for issues ranging from tobacco advertising to cigarette smuggling. The action was praised by many groups, but it is not apparent whether the United States will actually ratify the treaty; 40 governments must ratify the treaty for it to take effect, but only 9 have done so. With 5 million people around the world dying from tobacco-related causes each year, supporters hope the treaty will come into force. Among other actions, signatories must ban cigarette advertising, increase taxes on tobacco products, and require cigarette manufacturers to size health warnings to take up at least 30% of the package label.

## Where Does the Old Oil Go?

The United States generates approximately 1 billion gallons of used automotive, hydraulic, and cutting oils each year, 75% of which is resold untreated as a cheap industrial fuel. This practice leads to significant emissions of toxic metals including lead and cadmium, according to a 15 January 2004 report in *Environmental Science & Technology*. The report compared three ways of dealing with used oil: re-refining it, distilling it, and burning it untreated. The authors found the toxicity potential of using the untreated oil was 150 times greater for terrestrial ecosystems and 5 times greater for humans. Development of better oil filters for cars and less frequent oil changes can greatly reduce the volume of used oil.

## Figures and Tables

**Figure:**
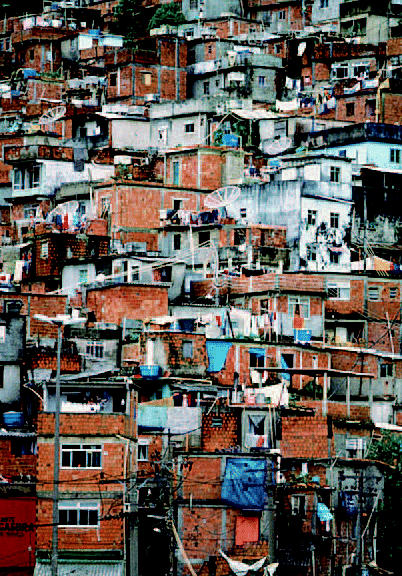


**Figure:**
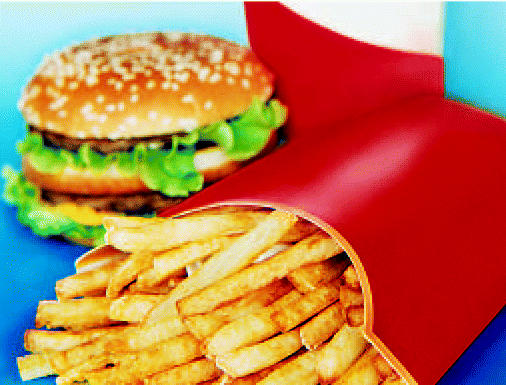


**Figure:**
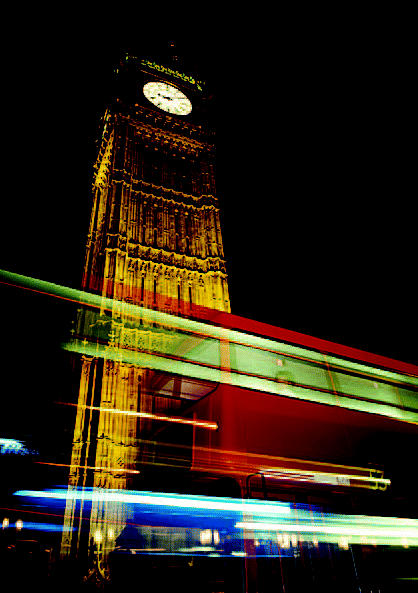


**Figure:**